# Cuffless blood pressure monitoring from a wristband with calibration-free algorithms for sensing location based on bio-impedance sensor array and autoencoder

**DOI:** 10.1038/s41598-021-03612-1

**Published:** 2022-01-10

**Authors:** Bassem Ibrahim, Roozbeh Jafari

**Affiliations:** 1 Department of Electrical and Computer Engineering, Texas A&M University, College Station, TX USA; 2 Department of Biomedical Engineering, Texas A&M University, College Station, TX USA; 3 Department of Computer Science and Engineering, Texas A&M University, College Station, TX USA

**Keywords:** Diagnostic markers, Diagnostic markers

## Abstract

Continuous monitoring of blood pressure (BP) is essential for the prediction and the prevention of cardiovascular diseases. Cuffless BP methods based on non-invasive sensors integrated into wearable devices can translate blood pulsatile activity into continuous BP data. However, local blood pulsatile sensors from wearable devices suffer from inaccurate pulsatile activity measurement based on superficial capillaries, large form-factor devices and BP variation with sensor location which degrade the accuracy of BP estimation and the device wearability. This study presents a cuffless BP monitoring method based on a novel bio-impedance (Bio-Z) sensor array built in a flexible wristband with small-form factor that provides a robust blood pulsatile sensing and BP estimation without calibration methods for the sensing location. We use a convolutional neural network (CNN) autoencoder that reconstructs an accurate estimate of the arterial pulse signal independent of sensing location from a group of six Bio-Z sensors within the sensor array. We rely on an Adaptive Boosting regression model which maps the features of the estimated arterial pulse signal to systolic and diastolic BP readings. BP was accurately estimated with average error and correlation coefficient of 0.5 ± 5.0 mmHg and 0.80 for diastolic BP, and 0.2 ± 6.5 mmHg and 0.79 for systolic BP, respectively.

Hemodynamic parameters, consisting of heart rate, stroke volume, cardiac output, peripheral resistance of blood vessels, and blood pressure (BP) are fundamental biomarkers that determine the proper function of the cardiovascular system. Abnormalities in hemodynamic parameters result in cardiovascular diseases (CVDs), which involve disorders and diseases related to the heart as well as blood vessels. According to the World Health Organization, CVDs are the leading causes of global death, and responsible for around one-third of all deaths across the world. In addition, according to the Harvard School of Public Health, the estimated global cost of CVDs was $863 billion in 2010, and is expected to reach $1044 billion in two decades^1^. As a result, in order to decrease the mortality rate and to reduce the economic burden of CVDs, prognostic and preventive policies are of paramount importance.

BP is a key risk factor in the prediction of CVDs^2^, which can be measured and monitored through non-invasive techniques. In order to effectively diagnose and manage cardiovascular events; continuous, passive, and frequent monitoring of BP is significantly required. Due to a variety of human activities throughout the day and different physical and mental reactions to daily incidents, daytime BP measurements are insufficient as a reliable predictor of CVDs. On the other hand, at nighttime and during sleep, BP readings demonstrate a more precise measurement as they reflect an individual’s true cardiovascular circulatory health^3^. Therefore, nighttime BP monitoring plays a more prognostic role than the daytime measurement. Sphygmomanometers and oscillometric methods are the current BP measurement systems that operate using an inflatable rubber cuff wrapped around the arm. Even though these cuff-based systems are widely used in clinical routines; they are bulky and obtrusive, and are appropriate only for infrequent readings^4^. In addition, vital nighttime BP monitoring is not supported by cuff-based systems. Even with automated BP reading systems, the cuff’s inflation and deflation may suddenly wake up the individual at night and alter the nighttime reading. Moreover, while automated BP systems drop the necessity of in-clinic and by-specialist measurements, their accuracy is unknown^5^. As a result, a cuffless BP monitoring system with a wearable form factor is essential to achieving continuous BP monitoring during day and night.

The characteristics of blood pulsation in blood vessels have received extensive consideration in the implementation of cuffless BP monitoring systems^6–11^. Pulse transit time (PTT) is defined as the time taken by the pulsatile blood flow to travel through an artery between two fixed points during each cardiac cycle. Based on Moens–Korteweg equation, PTT is highly correlated with BP^12^, and in fact, is inversely proportional to BP. In addition, PTT can be derived from simultaneous monitoring of photoplethysmogram (PPG) signal, which detects the changes of blood volume in arteries, and electrocardiogram (ECG) pulse, which acquires the cardiac bioelectrical activity in terms of impulses over time. PTT is approximately measured as the time difference between each R-peak of the ECG signal and a characteristic point on the PPG pulse^13–16^. Also, seismocardiogram (SCG) which detects the local vibration of the thorax wall can be employed as an alternative for the ECG^17^. However, the existing systems suffer from three major issues. First, monitoring of either ECG or SCG signals requires a pair of patches on the chest, which in combination with the distal pulse arrival PPG sensor on either arm or wrist cannot be integrated into a wearable device with a small form factor. Moreover, the PTT measured based on ECG R-peak includes the time interval from the start of ventricular depolarization until the moment of aortic valve opening, called the pre-ejection period (PEP). More specifically, the delay period between the ECG R-peak and PPG signal is known as pulse arrival time (PAT) which consists of PTT and PEP. However, PEP is not correlated with BP and besides, it cannot be measured, independently. In addition, in the case of patients with cardiovascular disorders, the PEP becomes even more significant and generates a source of error for the estimation of BP. Therefore, the BP estimation through the PTT measured based on ECG signal inherently carries a significant level of error^18–21^. For accurate BP monitoring, we need to focus on the arteries which lie deep in the tissue and its pulsatile activity is highly correlated to BP. However, the PPG sensor relies on the light signal that has a limited penetration depth of few millimeters which can not reach the arteries at the wrist or the arm. Instead, the PPG sensor capture pulsatile activities in capillaries, which do not accurately reflect on BP. Therefore, it is important to rely on a new cuffless BP measurement method that does not depend on ECG and PPG and provides accurate monitoring of blood pulsatile activity for more accurate BP readings from small-form factor devices.

In this work, we propose a new wrist-worn method for cuffless BP monitoring based on bio-impedance (Bio-Z) sensors that monitor the pulsatile activity of the wrist arteries with high accuracy and reliability to provide high fidelity BP estimation. The Bio-Z sensing method is a non-invasive technique that in general, can detect the static and dynamic behavior of body composition and live tissues. The Bio-Z signal is measured by injecting alternating current (AC) signals into the body via a pair of electrodes and then, sensing the potential difference on another pair. The amplitude and phase of the measured voltage signal are modulated by the tissue impedance at the sensing site. One major advantage of Bio-Z sensing is the deep penetration of the current signal in the tissue and hence, can detect the blood pulsatile at the depth of wrist arteries from the changes in bio-impedance. Therefore, Bio-Z signals can provide more accurate BP readings compared to PPG sensors (Figure S3). However, there are multiple challenges with BP estimation from wrist-worn devices based on local blood pulsatile sensors placed on a small area at the wrist. The small-form factor devices limit BP estimation to a small area on the peripheral wrist arteries which results in low sensitivity of pulse features to BP. In addition, the sensing location relative to the artery is critical to BP estimation because it affects the morphology and quality of the blood pulsatile signals. Therefore, we created a paradigm upon an array of Bio-Z sensors to accurately detect the morphology of arterial pulsatile for better BP estimation. The concept of sensor array allows the detection of the pulsatile activity of both radial and ulnar arteries simultaneously which provides comprehensive BP features for a more accurate estimation of BP. In addition, the Bio-Z sensor array is easily configurable by changing the electrodes for current injection and voltage sensing on the fly to select the sensing location close to the artery that has the highest signal quality of blood pulsatile. The electrode selection can be based on the quality metrics for collected Bio-Z signals, e.g. peak-to-peak amplitude and signal-to-noise (SNR) ratio, which can be defined at the processor of the hardware connected to the wristband. Besides, Bio-Z sensors are low cost and low power; therefore, they are easy to build and can be used for a large array of sensors.

In this study, we use the Bio-Z sensor array in a novel method to accurately estimate the true pulsatile activity of the artery independent of the sensing location. The BP models that are trained by features extracted from the estimated arterial pulse signal provide consistent and reliable BP predictions with different sensing locations. This calibration-free method for the sensing location relies on combining the pulse signals from multiple sensing locations to reconstruct the true arterial pulse signal independent of the sensing location. The reconstruction method depends on an autoencoder which is an unsupervised machine learning algorithm that can compress a high-dimension input into its lower dimension representation. The estimated lower dimension signal is equivalent to the target pulsatile activity. The autoencoder is implemented using a convolutional neural network (CNN) which can effectively learn the transfer function between the artery and each sensor based on the training data of Bio-Z pulse signals without any labels. As a result, the autoencoder can reconstruct the artery’s pulsatile signal which is used for BP feature extraction and predictions. In addition, we propose a new additional set of features that focus on the reliable representation of the significant parts in the pulse morphology for more accurate BP prediction. The new set of features include the histogram of the pulse amplitude in addition to the complete characteristics of the dicrotic notch which is the secondary peak point in the middle of the heartbeat pulse morphology. We show that these features are highly correlated with BP and can significantly improve the BP predictions.

In this work, we show the development of the hardware and signal processing algorithms for a cuffless BP system based on a wrist-worn device. The system includes an array of 6 Bio-Z sensors activated from 3 electrode columns in the wristband of the electrode array as shown in Fig. 1(a). The wristband is connected to our custom multi-channel Bio-Z sensing hardware for high-resolution Bio-Z sensing. The proposed CNN autoencoder reconstructs the radial arterial pulse signal from the raw pulse signals from the 6 Bio-Z sensors. This study demonstrates using AdaBoost regression models based on the features extracted from the arterial pulse signal to estimate SBP and DBP based on the significant BP features in the morphology of the estimated arterial pulse signal. We show in this work that the Bio-Z sensor array combined with the CNN autoencoder method can estimate reliable pulsatile activity that improves the BP estimation accuracy at different sensing locations compared to the use of a single Bio-Z pulse signal.

**Figure 1:**
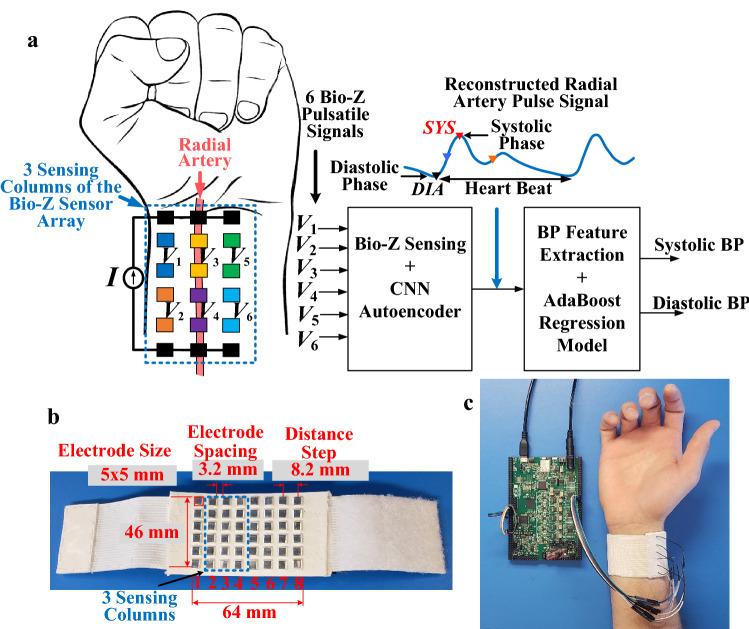
(**a**) The proposed wrist worn device for the cuffless blood pressure (BP) monitoring based on 6 Bio-Z signals sensed from 3 columns of the (Bio-Z) sensor array. A convolutional neural network (CNN) autoencoder algorithm is used to reconstruct the arterial pulse signal from the sensor array. The systolic and diastolic BP are predicted based on AdaBoost regression model trained by BP features extracted from the estimated arterial pulse signal. (**b**) The implemented wrist-worn sensor array consisting of 6 × 8 silver electrodes. The size of each electrode is 5 mm × 5 mm and the spacing between each two adjacent electrodes is 3.2 mm, (**c**) Integration of the wristband to the Bio-Z sensing hardware through our designed Bio-Z XL PCB for detecting the bio-impedance signals.

## Results

The human subject experiments for BP estimation were performed with a total of N = 4 subjects with age range from 20 to 25 years. In order to train and test the subject-specific BP models, we collected the wrist Bio-Z data from our sensor array simultaneously with reference BP data from a standard BP device. The Bio-Z signals were measured by the electrode array wristband with a size of 46 × 64 mm for the electrode array that was connected to the Bio-Z sensing hardware implemented in our custom Bio-Z XL board as shown in Fig. 1(b). The wristband includes 6 × 8 array of silver electrodes with a size of 5 × 5 mm and 8.2 mm center-to-center spacing between electrodes as shown in Fig. 1(c). The electrode array was placed at the bottom side of the wrist to be close to the radial and ulnar arteries of the wrist for effective sensing of the arteries' pulsatile activity. Three adjacent columns of the electrode array were utilized for Bio-Z sensing to measure 6 Bio-Z channels (*V*_1_ to *V*_6_) from different sensing locations around the radial artery.

Data collection was done during applying significant BP changes temporarily to evaluate our methods in predicting extreme BP changes. The experiment relied on repeating multiple trials of BP maneuvers that include elevation of BP temporarily above the normal level followed by BP recovery to normal values. The BP maneuvers we utilized for this study were based on using handgrip exercise and cold pressor test to elevate BP. In addition, we collected data at different sensing locations to evaluate the performance of the proposed BP estimation algorithms by changing the sensing location after training the BP model.

**Figure 2:**
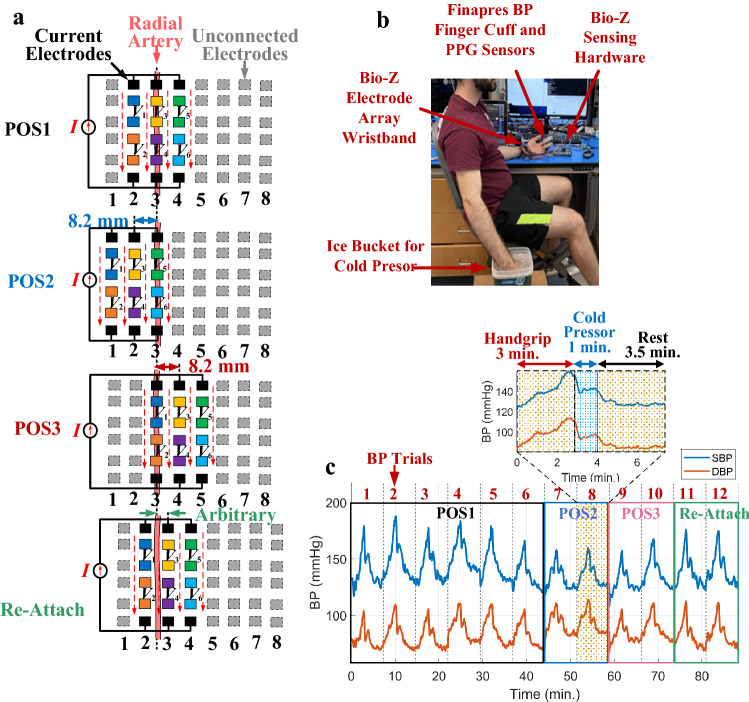
(**a**) The electrode configuration for the 6 Bio-Z signals from 3 sensing columns that are collected at four different sensing locations which are POS1, POS2, POS3 and Re-Attach with the illustration of the used electrode columns and the distance relative to the radial artery for each sensing location. (**b**) The data collection setup showing the participant placing his left arm on the bench with the attached Finapres BP finger cup, PPG finger clip and our Bio-Z electrode array wristband that is connected to our custom Bio-Z sensing hardware (Bio-Z XL) while the participant’s right arm is placed in the ice bucket for the cold pressor that followed hand grip exercise using the same hand. (**c**) The systolic BP and diastolic BP change during around 90 min of the experiment of a single participant which includes 12 repeated trials to increase BP by 3 min of handgrip exercising, 1 min of cold pressor test followed by 3.5 min at rest for BP recovery for each trial. The participant in the photo gave his consent for his photo to be used for publication.

In order to provide sufficient data for BP model training, we collected 90 min of Bio-Z and BP data with around 6000 heartbeats from each participant divided among 12 trials of BP maneuvers and 4 sensing locations called POS1, POS2, POS3 and Re-Attach. The first 6 BP trials were measured at the initial sensing location POS1 from columns 2,3, and 4 with the middle column 3 aligned with the radial artery as shown in Fig. 2(a).

**Figure 3:**
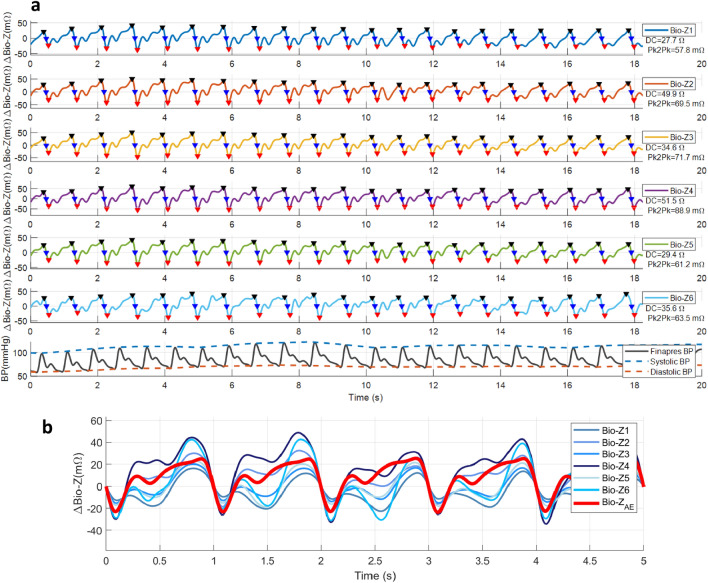
(**a**) An example of the experimental data collected by the Bio-Z XL board and the electrode array wrist band for the 6 sensing locations at the wrist around the radial artery simultaneously with continuous BP signal from the reference Finapres BP device. The data illustrates the Bio-Z pulse signal (ΔBio-Z) after removing the DC component by the signal pre-processing algorithms and after characteristic points detection. The plot highlights the DIA peak (black), MS (blue) and SYS foot (red) points. The reference systolic and diastolic BP are shown as the interpolation of the peak and foot points of the continuous BP signal from Finapres. (**b**) The plot of the CNN autoencoder output of radial artery’s pulsation signal (ΔBio-Z_AE_ in red) which estimated from the input Bio-Z pulse signals (ΔBio-Z in blue) measured from different sensing locations at the wrist by learning the transfer function from the artery to each sensing location from the training data. The plot shows the high quality and consistency of the estimated arterial pulse signal compared to the input signals.

Figure 3(a) shows an example of the experimental data collected by the Bio-Z XL board and the electrode array wrist band for the 6 Bio-Z channels at POS1 at the wrist around the radial artery. The data illustrates the high-quality Bio-Z pulse signal (ΔBio-Z) for all channels after removing the DC component of static tissue impedance by the signal pre-processing algorithms. The plot highlights the DIA peak (black), MS (blue) and SYS foot (red) points for each heartbeat that are estimated by the characteristic points detection algorithms with high accuracy and consistency for different pulse morphologies. The plot shows the average DC component and peak-to-peak value of the pulse (Pk2Pk) over 2.5 min time segment for each signal that varies from 27.4Ω to 43.5Ω and from 46.0mΩ to 120.7mΩ respectively. The largest signal occurs in the middle column at sensor 4 because of its alignment with the radial artery and the higher current density at the middle column. In addition, Fig. 3(a) shows the reference BP signal from the Finapres device as measured simultaneously with the Bio-Z signals. The reference systolic and diastolic BP are shown for every heartbeat as the interpolation of the peak and foot points of the continuous BP signal from Finapres.

The next step in the proposed algorithms towards BP prediction is the estimation of the arterial pulse signal from the 6 input ΔBio-Z signals using the CNN autoencoder algorithm. The output signal from the CNN autoencoder, defined as ΔBio-Z_AE_, is the estimation of the pulsation in the radial artery based on filtering the input signals with CNN weights that are learned from the ΔBio-Z data and represent the transfer functions between pulsation in the artery to the sensors on the skin. An example of the CNN autoencoder output ΔBio-Z_AE_ is shown in Fig. 3(b) compared to the 6 input ΔBio-Z signals. The estimated signal has high-quality pulse morphology that is consistent over multiple beats and is considered as the accurate arterial pulse signal. The BP prediction with regression models relies on the features extracted from this estimated arterial pulse signal. The effectiveness of the proposed method in arterial pulse estimation is assessed by the improvement that occurs in BP estimation by this signal compared to the baseline method that relies on the raw input ΔBio-Z pulse signals.

The data were collected from 4 healthy participants using our developed wristband and sensing hardware and were captured over 12 consecutive BP trials of 7.5 min for each trial as shown in Fig. 2(c). As a result, the measurements were conducted over a total period of 90 min for each participant. The DBP and SBP were estimated using separate regression models based on the ensemble learning method of AdaBoost, which builds the prediction by combining several weak learners' outputs through a weighted sum of different subsets of the training data set (See Supplementary Note 3). The BP AdaBoost model is trained by the BP features that are extracted from the wrist ΔBio-Z signals for each subject after processing with the CNN autoencoder. The ΔBio-Z signals are measured simultaneously with the reference BP signal measured from the Finapres BP reference device. We used subject-specific models that were trained for each subject based on the subject’s data to capture the unique arterial properties for each individual. The performance of the models was evaluated using the BP mean error (ME), standard deviation error (STD), root-mean-square error (RMSE), and correlation coefficient (R). We used three different training and testing methods to evaluate the BP prediction performance that varies in splitting the data between training and testing and the number of training iterations within each method.

The first method of 20-fold cross-validation trains and tests the model using POS1 data only by splitting the data into 20 folds with 19 folds (95% of POS1 data) for training and onefold (5% of POS1 data, 2.25 min) for testing. The performance of this method is evaluated by the average of all 20 folds. The second method evaluates the BP model performance for a longer continuous time segment of POS1 data by using leave one complete trial out cross-validation method. In this method, the POS1 data is splitted into 5 BP trials of POS1 data (37.5 min.) for training data and the remaining single BP trial of POS1 (7.5 min.) for the testing data. The third method evaluates the BP performance for different sensing locations and for longer time segments by training the model with the first 4 BP trials of POS1 data (30 min) and testing the model with the remaining 8 BP trials for the four locations (POS1, POS2, POS3 and Re-Attach) by taking the average of each 2 trial for each location which are 15 min. time segment. The arterial pulse signal (ΔBio-Z_AE_) is estimated for each location by four separate CNN models denoted by AE1, AE2, AE3 and AE4 for the locations POS1, POS2, POS3 and Re-Attach respectively. Detailed comparison between the training methods is presented in Table S2 and Figure S4.

**Figure 4:**
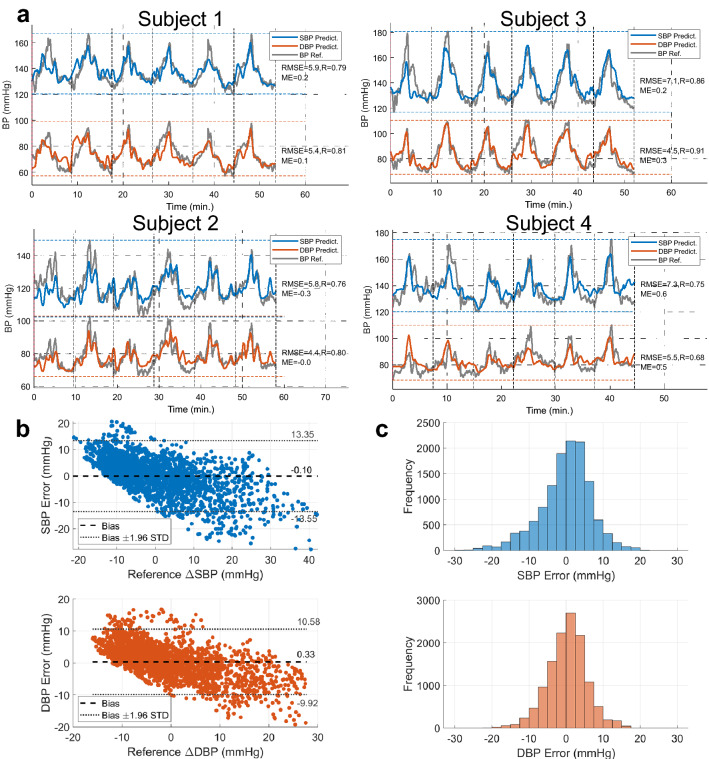
Bio-Z Array BP performance evaluation. (**a**) The plot of SBP (blue) and DBP (orange) predictions versus reference BP from Finapres (gray) over time for each subject for the first 6 BP trials (BP trials from 1 to 6) of POS1 using the proposed method with 20-fold cross validation. (**b**) The Bland–Altman plots of SBP and DBP estimation error versus reference BP from Finapres after removing the mean BP for all subjects using the proposed method with leave one trial out cross validation. (**c**) Histograms of SBP and DBP estimation error.

The BP performance for each subject using the first training method of 20-fold cross-validation is graphically illustrated in Fig. 4(a) (See more information in Table S3). The proposed method is tested for extreme BP values by introducing large changes in each subject BP using handgrip exercising followed by cold pressor. The DBP varies across the 4 subjects with an average range of 40.8 mmHg between a minimum and maximum values of 64.8 mmHg and 105.7 mmHg with STD of 2.3 mmHg, 4.6 mmHg and 4.8 mmHg, respectively. While the SBP varies with an average range of 53.0 mmHg between a minimum and maximum values of 114.9 mmHg and 167.9 mmHg with STD of 7.0 mmHg, 7.3 mmHg and 11.7 mmHg, respectively. The average BP performance using 20-fold cross-validation for the DBP is illustrated by an average RMSE of 5.0 mmHg and a substantial average correlation coefficient of 0.80 with respective insignificant STDs of 0.5 mmHg and 0.08 demonstrate high accuracy estimation of the DBP from bio-impedance features in comparison with the DBP acquired by the reference BP monitoring system. In addition, an average of the mean error (ME) of 0.2 mmHg shows the precision in BP estimation by our developed wrist-worn sensor array.

On the other side, an average RMSE of 6.6 mmHg with an average correlation coefficient of 0.79 and respective STDs of 0.7 mmHg and 0.04 were estimated for the SBP. In addition, the mapping algorithm resulted in an average BP mean value of 133.3 mmHg and an average ME of − 0.2 mmHg with STD of 7.6 mmHg and 0.3 mmHg, respectively. Figure 4(a) shows the estimated SBP and DBP plotted in comparison to the reference BP for the study participants for the time duration of 45 min. of POS1 data. The insignificant values of RMSE and ME along with the remarkable correlation coefficient illustrates consistency between the predicted SBP and DBP values and the reference BP measurements.

In addition, the breakdown of the contribution of all steps in the proposed algorithm including using AE only, AE with adding new features and finally adding the BP averaging are compared with the baseline method for 20-fold cross-validation as shown in Table S4. The performance of DBP and SBP estimation with the proposed method is compared by the baseline method. The baseline performance is calculated from the average of the two sensors in the middle electrode column which have the largest pulse signals and best performance among all the electrode columns. The proposed method with 20-fold cross-validation shows significant improvement in RMSE and correlation coefficient and each step in the proposed method is partially responsible for this improvement. For the 20-fold cross-validation method, using CNN autoencoder causes BP to improve compared to the baseline from 6.6 mmHg and 0.64 to 6.0 mmHg and 0.71 for the DBP and from 9.0 mmHg and 0.57 to 8.1 mmHg and 0.67 for the SBP. Using new proposed features with CNN autoencoder causes BP to improve to 5.7 mmHg and 0.74 for the DBP and 7.5 mmHg and 0.73 for the SBP. Finally using the complete proposed method by adding the BP averaging cause BP performance to achieve its maximum of 5.0 mmHg and 0.80 for the DBP and 6.6 mmHg and 0.79 for the SBP.

The training method of leave one trial out shows the effect of decreasing the training data and increasing the testing data to one complete trial. The BP performance of the proposed method using leave on trial out is shown in Table S5. The RMSE and correlation coefficient is 5.2 mmHg and 0.77 for the DBP and 6.9 mmHg and 0.76 for the SBP with only slight degradation compared to the 20-fold cross-validation method. The Bland–Altman plots and histograms of DBP and SBP errors are plotted in Fig. 4(b) and (c) respectively.

For the proposed method with leave one trial out cross validation, the DBP and SBP error distribution for the three BP error ranges under the thresholds 5 mmHg, 10 mmHg and 15 mmHg are 69%, 94% and 99% for DBP and 60%, 86% and 96% for SBP. These results show that the BP performance is consistent with grade A for both DBP and SBP according to the British Hypertension Society (BHS) standard^22^ (See Table S6).

The BP performance of the last method of testing on different locations using the proposed method compared to the baseline method is shown in Table S7. The third training method shows the effectiveness of the proposed method when training the model on POS1 data and testing the model on other positions POS2, POS3 and Re-attach. The baseline method has the best performance when the model is trained and tested on the same location of POS1 which is RMSE and correlation coefficient of 7.2 mmHg and 0.67 for DBP and 8.2 mmHg and 0.64 for SBP. The baseline method performs poorly when tested on different locations with RMSE and correlation coefficient of 9.4 mmHg and 0.50 for DBP and 11.4 mmHg and 0.47 for SBP for the average of the three locations. The proposed method improves the BP performance at different locations by RMSE and correlation coefficient of 7.4 mmHg and 0.72 for DBP and 10.2 mmHg and 0.62 for SBP with an average improvement of 34.1% in correlation coefficient and 15.9% in RMSE compared to the baseline method at different locations. The BP performance of the proposed method at different locations is close to the baseline method at the same location of POS1 by a factor of 92%. The degradation in BP performance in this case compared to the 20-fold cross-validation and the leave on trial out due to the reduction in the training data from 95% and 83.3% to 66.7% of POS1 data and the increase in the testing time segment from 2.25 min. and 7.5 min. to 15 min.

## Discussion

In this study, we demonstrated the feasibility and effectiveness of predicting systolic and diastolic BP values from the bio-impedance signals detected from the wrist’s radial artery. The signals are acquired through a non-invasive cuffless wristband with a flexible sensor array. A CNN autoencoder algorithm was proposed to estimate the arterial pulse signal from the input six Bio-Z signals measured from the wrist. In addition, a BP mapping algorithm based on a subject-specific AdaBoost regression model was developed and trained by the bio-impedance features extracted from the estimated pulse signal. The CNN autoencoder is required to be trained for each individual to capture the unique mapping between the sensing location and pulsatile features for each person. However, our CNN autoencoder has the advantage of being an unsupervised machine learning method that learns the CNN weights continuously and online from the input data without the need for labels or specific calibration data. This ensures that the CNN model weights keep updated with any change in the sensing location.

The proposed cuffless BP system was shown its effectiveness in predicting BP for continuous-time segments up to 15 min. and for large BP changes up to 53.0 mmHg with acceptable accuracy. The BP prediction methods were reliable for two different types of BP maneuvers which are exercising and cold pressor. In addition, the effect of changing the sensing location on the BP prediction performance was shown for the first time and we were able to improve the results by 34.1% in correlation coefficient and 15.9% in RMSE by using our proposed methods of CNN autoencoder to estimate the arterial pulsation independent on the location in addition to the proposed new features and BP averaging technique. For additional improvements for the BP predictions, the sensor array can be extended to add the signals from the ulnar artery to the current signals from the radial artery. We only used 3 columns from the 8 columns of the sensor array in our wristband. Additional 3 columns can be used around the ulnar artery to utilize the full array band and to generate an estimation of the arterial pulse at the ulnar artery.

Since the vascular properties of ulnar and radial arteries vary from one subject to another, a general model cannot be utilized. In this manuscript, we showed the variance in the arterial pulse morphologies among the participants, Therefore, a regression model based on AdaBoost technique was used for each participant. AdaBoost showed a higher correlation coefficient between the estimated and the reference BP and a lower RMSE in comparison with the Support Vector, Random Forest, Linear, Gradient Boosting, and Decision Tree regression models^23^. Since mapping the bio-impedance features to BP values is a nonlinear problem, the AdaBoost algorithm provides the most accurate results.

The time length of bio-impedance data collection has a direct impact on the data optimization process and the accuracy of BP estimation from the extracted characteristic points. We showed that longer training data will increase the correlation coefficient between the estimated and the measured BP and will decrease the RMSE between them. Our current hardware system does not have a wearable form factor and best-case scenario, the time length of collected data will be limited to a few hours which can be improved in the future version of the system. In order to facilitate long-term bio-impedance data collection and to continuously read BP, this system can be improved by implementing a wearable version with the form factor of a smartwatch. Such a system can acquire the required amount of data as the input to a new complex training model such as deep neural networks (DNN) and as a result, a generalized model for mapping bio-impedance extracted features to BP values can be investigated. In addition, the long-term accuracy of BP predictions over multiple weeks can be evaluated by collecting data from the same subject over long-period of time to evaluate the performance of BP model over time. Another possible improvement is minimizing the size of the calibration data that is required to train the subject-specific BP model for a new subject through transfer learning methods that utilize data from previous subjects. The long-term and continuous reading of BP opens a new horizon in the prognosis and prevention of cardiovascular disorders and diseases, and potentially impacts the mortality rate of CVDs.

## Methods

### Background

In one of our previous works, we demonstrated the proof of concept for a cuffless BP estimation based on four Bio-Z sensors using subject-specific regression models^23^. We placed two pairs of sensors on the radial and the ulnar arteries. The pulse morphology is captured from the significant characteristic points of each heartbeat in the estimated Bio-Z pulsatile activity. The characteristic points are the basis for our BP estimation algorithm, which are highly correlated with the changes of BP and translate the Bio-Z as a function of time to BP. A regression model is developed based on an Adaptive Boosting (AdaBoost) meta-algorithm which takes the features extracted from the characteristic points of the pulse signals, and through subject-specific model parameters, provides a precise estimation of systolic BP (SBP) and diastolic BP (DBP) readings. The subject-specific BP models are essential to capture the individual properties of the arterial blood pulsation that varies significantly from one person to another. We showed that using pulsatile activity from Bio-Z signals and using features extracted from multiple sensors at different arteries improve BP estimation compared to a single sensor. However, the Bio-Z sensors were based on conventional Ag/AgCl wet electrodes, which due to lack of wearability and dryness of the contact surface gel, are not appropriate for long-term data collection. In this work, we present an electrode array consists of small 48 built-in silver electrodes embedded in a 2-dimensional matrix of 6 by 8 that are robust against the aforementioned issues and demonstrate constant skin–electrode impedance with negligible fluctuations over time. Accurate mapping of Bio-Z pulses to BP substantially depends on accurate detection of dynamic time-dependent behavior of the wrist arterial pulsatile. Therefore, the wrist-worn sensor array is designed in a flexible wristband, which conforms with the wrist shape and provides a robust contact with the skin for reliable current injection and voltage sensing.

Our previous work was constrained by placing the 4 sensors precisely on the wrist arteries for the whole study after detecting the location of the arteries. The fixed sensing location provided low-error BP predictions because the BP models were trained and tested at the same sensing location based on consistent pulse signals. However, the sensor in a wrist-worn device changes its location frequently due to user movements and placing the device at different locations. Each sensing location provides different morphology for the pulse signal based on the tissue transfer function between the artery and sensor at the skin. Therefore, the change in sensing location affects the measured pulse signal and the BP models. As a result, BP models behave poorly by changing the sensing location. The solution to this problem can be expensive and complicated by calibrating the BP models for sensing location by collecting data from multiple locations and training multiple BP models for all possible locations. Then for BP predictions, the sensing location is detected to select the suitable BP model for predicting BP as a function of the sensing location. This approach requires collecting a lot of data at different sensing locations which is inconvenient to the user. In addition, complicated algorithms are required for detecting the sensing location and predicting BP. Therefore, we propose in this study a new simple and reliable method for accurate BP detection independent of the sensing location without the need for complex calibration algorithms for the sensing location.

### Introduction

The proposed method for cuffless BP measurements from a wrist-worn device rely on using small-form factor of non-invasive sensors that measure blood pulsatile activity from the arteries and transform them into BP models using regression models. The sensors need to be placed as close as possible to the artery for accurate and consistent measurement of the arterial pulsation that results reliable BP estimation. The change of sensing location away from the artery results in changes in pulse signal morphology and BP results. These changes are significant for the small-form factor sensors that are integrated into a wrist-worn device because they suffer from frequent position displacements on the wrist due to user movements of the arm and when the user takes off the device and re-attaches it to the wrist at a different location. These continuous displacements affect the measured pulse signal and the accuracy of BP estimation.

The arterial pulsation sensing can be modeled by source signal (*Y*) that represents the arterial pulse located deep inside the tissue at the location of the artery. The sensor placed on the skin measures the signal *V* which is the output of the transfer function (*h*) with the input is arterial pulsation *Y* that represents the effect of the tissue and the distance between the sensor and the artery as shown in Figure S5(a). Therefore, transfer function (*h*) changes for different locations of the sensor on the skin relative to the artery’s location which causes changes in the sensor output signal (*V*). Since, the BP estimation relies on the morphology of the pulse signal, the changes occur in the sensor output with location increase the BP error. In order to reduce the effect of the sensing location on the estimated signal, we propose using multi-sensor pulse signal estimation from multiple locations around the target artery instead of single point measurement as shown in Figure S5(b). Each sensor output is a function of the source arterial pulse signal with the function varies with sensing location. The estimated multi-sensor pulse signals are used for the reconstruction of the arterial pulse autoencoder. The autoencoder is an unsupervised machine learning algorithm that finds the lower-dimension representation of the high-dimension input signals. This method provides accurate pulsatile activity of the artery independent of the sensing location which improves the BP estimation at different locations.

In this work, the human subject experiments for BP estimation were performed under the approval of the Institutional Review Board (IRB) of the University of Texas A&M (IRB no. IRB2017-0335D). All research was performed in accordance with the IRB guidelines and regulations. All participants provided written informed consent to take part in the study.

### Arterial pulse estimation using autoencoder

Based on the explained sensing model, each pulse signal from the sensor output *X*_i_ at the skin is related to the source signal of the pulsatile activity *Y* at the artery deep inside the tissue through the discussed transfer function of a filter *h* that is defined by certain weights *b*_n_ and depends on the sensing location. Our objective is to reconstruct the hidden arterial pulse signal *Y* from the measured pulse signals from multiple sensors outputs *X*_i_ at different sensing locations for *i* = 1 to *K* where *K* is the number of sensors.

We propose using the unsupervised machine learning algorithm of autoencoder to estimate the arterial pulse signal *Y* from the input pulse signals *X*_i_. The autoencoder is capable of estimating a lower dimension representation, called the code, from the higher dimension inputs. The autoencoder consists of an encoder network that encodes the inputs from the input layer into a lower dimension representation in the hidden layer which is decoded by the encoder network to reconstruct the inputs at the output layer as shown in Figure S6. The layers of the encoder and decoder networks are implemented as neural networks and their weights are estimated through the gradient descent optimization method to minimize the error between the input and output layers by minimizing the loss function which is the square error between the input and output layers. As the number of sensors *K* and the dimension of the input increases, the accuracy of the code estimation increases.

The autoencoder can accurately estimate the arterial pulse signal when the decoder network is equivalent to the transfer functions *h* that maps the *Y* at hidden layer to the measured pulse signals *X*_i_ and in this case, the encoder network represents the target reconstruction function that reconstructs the arterial pulse from the input observations. This goal is achieved by implementing the encoder and decoder networks as convolutional neural network (CNN) with a linear activation layer which consists of a window of weights that sweeps the input dimensions similar to the operation of the filter that model the pulse transfer function. The input pulse signals are divided into overlapping time segments which are considered the input samples for the training of the CNN networks of the autoencoder. After the gradient descent optimization and minimizing the loss function, the weights of the encoder CNN are used to estimate the arterial pulse signal from the input signals which is used to extract the BP features for BP estimation using the regression models.

### System overview

The proposed wrist-worn BP monitoring system relies on an array of Bio-Z sensors from multiple locations on the wrist. The Bio-Z sensor array consists of six channels of Bio-Z sensing bio-instrumentation that interface with the body through a flexible wristband that conforms with the skin and includes a 2D array of metal electrodes to provide good contact with the skin for high-quality signal monitoring. The Bio-Z hardware includes injection of small AC current and voltage sensing from multiple pairs of electrodes. The measured voltage signals are pre-processed to extract raw Bio-Z signal which is followed by pulse detection algorithms to extract the pulse signals, denoted as ΔBio-Z. Then, the proposed CNN autoencoder combines the multiple Bio-Z pulse signals to estimate the arterial pulse signal called ΔBio-Z_AE_ which is used for BP prediction. The next step includes the detection of the characteristic points of the pulse signals that are used to extract the BP features. Then, AdaBoost regression models are used to estimate systolic and diastolic BP after training by Bio-Z and reference BP data collected simultaneously as explained in the following sections as shown in Figure S8.

### Bio-Z sensing hardware

The wrist-worn sensor array consists of 48 dry silver electrodes, each with an area of 5 mm × 5 mm and center-to-center spacing of 8.2 mm between each two adjacent sensing points. An important aspect of the developed wrist-worn sensor array is that it is built based on dry electrodes. Conventional Ag/AgCl wet electrodes reduce the wearability of the wristband, and besides, as the conductive gel of the wet electrodes dries over time, the electrode–skin impedance increases drastically. The proposed wristband developed based on dry silver electrodes provides an electrode–skin impedance with minimum fluctuations over time and ensures the detection of arterial pulse with excellent signal quality and high SNR ratio. The selection of the sensor array specifications addressed different trade-offs between the signal quality and size of the array. We aimed to develop a dense array of electrodes that cover a large area of the wrist arteries by using a large number of electrodes and small spacing between them to provide a large number of Bio-Z signals that will improve the performance of the autoencoder reconstruction algorithm. However, the quality and signal-to-noise ratio of the Bio-Z signal is improved by increasing the spacing between electrodes and lowering the skin–electrode impedance by increasing the size and conductivity of the electrode. We selected the sensing area to cover the bottom part of the wrist for most people with a width of 64 mm. The height of the device was selected 46 mm to limit the device size below 5 cm to provide small-form factor device. These specifications allowed the development of 48 electrodes that are arranged in 6 × 8 array of electrodes that cover the wrist arteries in a small form factor device with good Bio-Z signal quality. This electrode arrangement allows the sensing of 2 Bio-Z signals per column by injecting current between the top and bottom electrodes and sensing voltage between the 2 middle electrode pairs. Therefore, our electrode array can support up to 16 simultaneous Bio-Z signals with 2 measurements per column that cover the radial and ulnar arteries at the bottom of the wrist. We explored different materials for electrodes such as wet Ag/cl electrodes and stainless steel. Silver has an outstanding electrical conductivity of 62.1 × 10^6^ Siemens/m and a very low electrical resistivity of 15.9 × 10^−9^ Ω.m, which makes it an ideal choice for sensing bio-impedance signals, easy implementation and practical for long-term usage in wearable devices. For future work, there are other promising materials for electrodes such as graphene which is ultrathin material that is conformal with skin to provide accurate and consistent signals for a long period of time with a skin-friendly electrode.

The 6 × 8 array of electrodes are embedded in a flexible wristband, appropriate for conforming with the wrist shape. The flexible wristband is made from Ecoflex rubber which is a durable and flexible silicone that conforms with the shape of the wrist to provide good contact with the skin for all silver electrodes. The height of the electrodes and the spacing between them are precisely controlled by placing the electrodes in a mold designed for the wristband with a fixed location and depth for the electrodes with wire connections that are covered by the Ecoflex silicone. The wristband is then connected to the Bio-Z sensing hardware through wires for bio-impedance signal detection. The wrist-worn sensor array and the integration with the hardware are shown in Fig. 1. The Bio-Z sensing hardware consists of the circuitry to generate and inject alternating current signals to the wrist skin at programmable frequencies and amplitudes, and the analog signal conditioning circuit to sense, amplify, and filter the detected differential voltage on each pair of electrodes.

The high accuracy and low noise sensing of blood pulsation play a critical role in the estimation of BP which relies on precise amplitude and time features extracted from these signals. The main challenge of the sensing hardware and signal processing is the multi-channel detection of small Bio-Z variation (ΔBio-Z) ranges from 50 mΩ to 150 mΩ due to blood volume changes in the presence of large DC Bio-Z of around 50 Ω that represents the static tissue fluids. In addition, the signal measurement methods require high time resolution and continuous data acquisition for several minutes. The low-noise multichannel Bio-Z sensing hardware, the so-called Bio-Z XL, is explicitly designed to capture the slight variations in Bio-Z with high resolution. A custom-developed printed circuit board (PCB) is designed to provide simultaneous low-noise 6 Bio-Z sensing channels for this study, as shown in Fig. 1(c).

An ARM Cortex M4 microcontroller as the central processor, instructs and controls the essential functions of the system. The main functions consist of generating the programmable alternating current signal, sampling the sensed and amplified potential difference of voltage sensing electrodes and then, sending the digitized data to a stationary computer connected to the Bio-Z XL board through USB (See more details about Bio-Z circuit design in Supplementary Note 4).

In this study, three adjacent columns of the electrode array were utilized for Bio-Z sensing to measure 6 Bio-Z channels (*V*_1_ to *V*_6_) from different sensing locations around the radial artery. The Bio-Z current signal was injected in three columns between the electrodes at the top and bottom rows to provide the best current distribution for the three columns. The six bio-Z voltage signals were measured from the middle pairs in the three columns with 2 voltage measurements per column as shown in Figure S8. The measured voltage signals are combined by the proposed autoencoder method to reconstruct the arterial pulse signal independent of sensing location that improves BP prediction accuracy. The rest of the electrode columns are not used in this study, however, they can be added to our autoencoder method to include additional measurements from the ulnar artery to improve the accuracy of the reconstructed arterial pulse signal and improve BP estimation.

As the reference for BP data estimation, we employed BP monitoring by Finapres NOVA system. In fact, we collected the bio-impedance data through the wristband simultaneously with Finapres. The system continuously measures BP using a finger pressure cuff place on the middle finger that is calibrated by the brachial cuff. However, the data collected by our system and the BP measurements acquired by Finapres need to be synchronized. Therefore, since the Finapres system is equipped with continuous PPG monitoring and collects the PPG data using a finger clip along with BP, we added a PPG sensor to our Bio-Z XL device, as well. Based on a comparison between the unique pattern of inter-beat intervals of the two PPG signals, we can synchronize the BP estimated by our AdaBoost regression model and the BP measured by the Finapres system.

### Data collection procedures

In order to provide sufficient data for BP model training, we collected 90 min of Bio-Z and BP data with around 6000 heartbeats from each participant divided among 12 trials of BP maneuvers and 4 sensing locations called POS1, POS2, POS3 and Re-Attach. The first 6 BP trials were measured at the initial sensing location POS1 from columns 2,3, and 4 with the middle column 3 aligned with the radial artery as shown in Fig. 2(a). In order to consider the effect of different sensing locations on the BP estimation, we repeated the data collection at different sensing locations relative to the artery. We selected two fixed sensing locations by shifting the sensing electrode columns by 1 column to the left and the right of the radial artery without taking off the sensor band from the wrist. The selected electrode configuration provides displacement in sensing location by 8.2 mm to the left and the right of the radial artery which are defined as POS2 and POS3 (Fig. 2a). In addition to the fixed change in sensing location, we also considered an additional arbitrary sensing location by taking off the wrist band and re-attaching it again to the wrist at a random location such that the artery is between the electrode columns 2 and 4. The data was collected for 2 BP trials at the new sensing locations POS2, POS3, and Re-Attach which is enough data for testing the models that are trained for POS1.

During the experiment, the participant sits on a chair with his left arm on a bench at rest to minimize the effect of the motion artifacts on the Bio-Z and BP signals, and to keep the wrist at a constant height relative to the heart as shown in Fig. 2(b). Before placing the wristband on the participant’s wrist, we locate and mark the radial artery’s location using Huntleigh Dopplex MD2 Bi-Directional Doppler which measures the velocity of blood flow using a high sensitivity probe. The mark of the radial artery is used to align the sensing location of the Bio-Z electrodes in the wrist band with the artery’s location. The array band is placed on the wrist according to the initial sensing configuration POS1 which is based on electrode columns 2,3 and 4 with the center column 3 is aligned with the radial artery. In addition to the wrist-worn sensor array, we used the Finapres NOVA system, which continuously measures BP uses a finger pressure cuff placed on the middle finger of the left hand and automatically calibrated with the standard brachial pressure cuff. Finally, in order to avoid movement of the left hand which holds all the sensors, and therefore to prevent the motion artifacts and noise; the handgrip exercise and cold pressor tests are carried out using the right hand of the participant. The SBP and DBP readings are extracted from the maximum and minimum BP values at the peak and foot points of the reference BP signal for each heartbeat. The beat-to-beat SBP and DBP readings were smoothed by a moving average window of 20 heartbeats.

Each trial to change BP consists of 7.5 min. The trial starts by handgrip exercising for 3 min, followed by a cold pressor test for 1 min by immersing the right hand in an ice water container, and ends by 3.5 min at rest. During each trial, starting from normal BP, an increase in BP occurs gradually with handgrip until it reaches its peak value by the end of the 3 min of the exercise. After that, BP starts to decrease and the cold pressor jumps in to slow down the drop in BP by trying to elevate the BP again. After that, the BP recovers back slowly to normal BP during the resting period as shown in Fig. 2(c).

The choice of handgrip exercise compared to other exercising methods such as cycling or running on a treadmill provides a large enough BP increase of about 50 mmHg above normal BP with minimum wrist movements to decrease motion artifacts in the collected data. We chose the cold pressor test to follow handgrip exercise in order to include different physiological mechanisms of BP changes from exercising for more general BP models. Also, BP drops suddenly in healthy subjects after handgrip exercise; therefore, the cold pressor helps to keep BP high for a longer time. This is preferable in order to provide more data points at high BP for better training and testing of the BP models. The handgrip exercise and cold pressor test are the best BP maneuvers that can change BP with minimal increase in heart rate so that the model is focused on BP changes rather than heart rate changes. In addition, these 2 BP maneuvers can be performed by participants in the lab with a high degree of repeatability in BP profile from one iteration to another among all subjects.

Since the proposed data training model is subject-specific, a solid and reliable BP reference reading is inevitable. With respect to this requirement, we used Finapres NOVA system, which uses the standard brachial pressure cuff for self-calibration and continuously measures BP using a finger pressure cuff placed on the middle finger. The Finapres reference system has the U.S. Food and Drug Administration (FDA) approval and besides, has received significant attention as a reference device for continuous BP measurements^23–26^.

### Bio-impedance signal pre-processing

The Bio-Z signal processing is responsible for extracting the Bio-Z blood pulsation signal, defined as (ΔBio-Z), from the raw Bio-Z signal. The raw Bio-Z signal includes the ΔBio-Z pulse signal superimposed over a slowly-varying Bio-Z DC component that corresponds to the static tissue impedance. The most prominent feature in the raw Bio-Z signal is the sharp edge between the systolic and diastolic points repeated each heartbeat with the max. slope point in the middle of the edge. The DC component of Bio-Z is estimated from the interpolation between max. slope points detected beat-by-beat. The max. slope points are detected from the lower peaks of the first derivative of Bio-Z constrained by a minimum peak distance of 0.57 × (1/*HR*) and a minimum peak height of 35% of the lower peak envelope. The *HR* is the average heart rate of the processed time segment of 2.5 min calculated from the frequency of the most prominent peak of the signal power spectral density. The lower peak envelope of the raw Bio-Z signal is determined using spline interpolation over local maxima separated by at least 0.65 s. Then, the ΔBio-Z pulse signal is extracted by subtracting the estimated DC component of Bio-Z from the raw Bio-Z signal.

### CNN autoencoder

For generalization, we assume we have *K* sensor signals that will be combined with the CNN autoencoder to reconstruct the arterial pulse signal denoted as ΔBio-Z_out_ at the intermediate layer which the encoder output.

In order to arrange the input data for the CNN autoencoder, each pulse signal of ΔBio-Z is divided into separate heartbeats that have different amplitude and number of samples according to the heartbeat duration. The number of samples per heartbeat is equalized and downsampled to *L* samples for each heartbeat in the dataset per subject by interpolation of each heartbeat at new *L* time samples with equal time steps that span each heartbeat. The downsampling of the signals helps in reducing significantly the number of autoencoder parameters. Consequently, all the heartbeats are normalized in duration with the same time grid. In addition, the amplitude of the heartbeats is normalized by dividing the amplitude of the whole heartbeat by its peak-to-peak amplitude, so that the amplitude of all pulses has the same amplitude range.

The input K heartbeats sequence is divided into time segments of N heartbeats with a time step of 10% of the heartbeat period *L*. Then, each time segment is arranged in a 3D array of N heartbeats *N* × *K* × *L* with *N* is the number of heartbeats, *K* is the number of sensors and *L* is the length of the pulse. This 3D array is the input to the autoencoder as shown in Figure S9. The encoder CNN network consists of a 3D convolution window with dimensions of *N* × *K* × F and stride of 1, where *F* is the filter width as a segment within *L*. The decoder network consists 1D CNN network repeated *N* × *K* times with a convolution window size of *F* and stride of 1. All CNN networks have a linear activation layer and zero biases to match the linear filter model that was adopted for the transfer function from arterial pulse to the sensor measurement. The total parameters to be trained for the autoencoder using this implementation is 2 × *N* × *K* × *F*. The CNN autoencoder is implemented with Keras functions in Python. The autoencoder is trained using Adam optimizer as a stochastic gradient descent algorithm with mean squared error as loss function. In order to ensure the continuity of the final encoder output ΔBio-Z_out_, the encoder CNN is applied again after training with the input data as the concatenation of all the 3D arrays of the input data along the time axis. In addition, the encoder CNN coefficients are upsampled to the original heartbeat sampling rate and reapplied on the original heartbeat data before downsampling in order to generate the encoder output ΔBio-Z_out_ in the original high sampling rate to provide high time resolution in the next steps of point detection and feature extraction. In this study, the CNN autoencoder parameters are selected according to Table S8.

### Characteristic points detection

The dynamic cardiac activity of ΔBio-Z pulse signal is characterized by six characteristic points each heartbeat, consisting of diastolic peak (DIA), maximum slope (MS), systolic foot (SYS), inflection point (IP), dicrotic peak (DP) and dicrotic notch (DN)^27^. The selected points can completely abstract the blood pulse morphology and describe the main rising and falling edges of the heartbeat as shown in Figure S10(a). On every heartbeat, the ΔBio-Z signal descends from the first peak to the first foot, which indicates a sudden increase in the blood volume as the pressure pulse arrives at the sensing point on the wrist. The ΔBio-Z peak DIA and foot SYS points represent the diastolic and systolic phases of the BP pulse, respectively. Furthermore, due to higher vascular resistance, the back reflection of the BP pulse results in the second smaller dicrotic peak and notch in the middle of the heartbeat.

The MS point of ΔBio-Z corresponds to a foot point in the first derivative of the ΔBio-Z signal and a zero crossing in the second derivative of the ΔBio-Z signal. The DIA and SYS points are calculated from the first derivative of ΔBio-Z signal by the two zero-crossing points adjacent to the foot point. The IP point acts as the MS point at the second smaller falling edge in the same heartbeat. The IP point is calculated from the zero crossings in the second derivative of ΔBio-Z signal that follows the MS zero crossing in time in the same heartbeat. Also, the IP point corresponds to a secondary foot point in the first derivative that follows the main foot of the MS point. Similar to the DIA and SYS points, the DP and DN points are calculated from the two zero-crossing points adjacent to the IP second at the foot point of the first derivative of ΔBio-Z signal. The coordinates of the six characteristic points in terms of time and amplitude are the parameters employed to determine BP features. An example of the detection of the characteristic points of the ΔBio-Z based on the first and second derivatives is shown in Figure S11. The plot shows the point detection algorithm can accurately detect all the characteristic points for six heart beats in the presence of variations in the pulse morphology from beat to beat.

### Bio-impedance BP features extraction

Based on the DIA, MS, SYS, IP, DN, and DP characteristic points, the features extracted from the bio-impedance pulse signal are categorized into sets of time, amplitude, area, dicrotic and histogram. The time intervals between DIA and MS, SYS, and IP are the time parameters for each signal normalized by the inter-beat interval (T_IBI_) which is the period of the heartbeat between successive MS points. Besides, the amplitude parameters for each bio-impedance pulse are the amplitude differences from DIA to MS and IP points normalized by pulse foot to peak amplitude A_SYS_. Moreover, the area under the bio-impedance signal from DIA to the MS, SYS, and IP points represent the area parameters for each signal normalized by the full pulse area. These sets of features were used in previous work and we introduce a new set of dicrotic features in this work. The dicrotic set of features are added to include the amplitude and time between dicrotic peak and notch normalized by A_SYS_ and T_IBI_ respectively which are highly correlated with BP changes. The final set of features is a new proposed feature for the pulse amplitude histogram based on the population of 5 amplitude bins which are the division of the normalized pulse amplitude into 5 equal intervals. The time, amplitude, area, dicrotic and histogram parameters are shown in Figure S10(b), (c), and (d) and explained in Table S9.

The aforementioned features are substantially related to the cardiac output and the arterial stiffness of the wrist arteries and so, are highly correlated with BP. The time interval between the systolic foot and the inflection point measures the arterial stiffness, and besides, the area under the curve represents the total peripheral resistance of the blood vessel^28^. In addition, the ratio between the amplitudes of the systolic foot and inflection point relative to the diastolic peak determines the intensity of the reflection wave. In this regard, the features can accurately model the vascular properties of the wrist arteries and will be the basis for estimating the DBP and SBP.

### BP prediction model

An advanced regression model is employed to translate the features extracted from bio-impedance signals to BP. Besides, since the DBP and SBP rely on different features, a separate regression model is utilized for each of them. In order to improve the accuracy of BP estimation, the individual variations of each participant’s vascular properties are acquired. Therefore, the subject-specific models are trained for each participant in our study using a limited number of training window samples. AdaBoost is the regression model we used for our BP estimation. It is a meta-algorithm which by training a sequence of weak models through a weighted sum of different subsets of the training data set, improves the prediction power of the algorithm and boosts the performance of decision trees. In fact, AdaBoost is an ensemble technique that attempts to convert a set of weak classifiers into a strong one. The hyper-parameters of the AdaBoost models consist of the number of the decision trees and the tree depths, which were selected as 32 and 8, respectively. For each model, the tree depth with the minimum testing error is selected to provide the best model complexity that avoids both overfitting and underfitting. The Bio-Z and BP data are splitted by different configurations as explained in the Experimental Results section.

### Ethical statement

The human subject BP measurements were performed under the approval of the Institutional Review Board of the University of Texas A&M (IRB no. IRB2017-0086D).

## Supplementary Information

Supplementary Information.

## Data Availability

The data supporting the findings of this study are available within the paper. The associated pre-processed raw data is available and can be shared with interested parties upon reasonable request.

## Code availability

The custom codes used for ML and data processing are available from the corresponding author upon reasonable request.
