# Supplementary material for: Cuffless blood pressure monitoring from a wristband with calibration-free algorithms for sensing location based on bio-impedance sensor array and autoencoder
Source: Sci Rep. 2022 Jan 10;12:319. doi: 10.1038/s41598-021-03612-1 (PMC8748973; doi:10.1038/s41598-021-03612-1)
Supplement: Supplementary file 1 — Supplementary Information. [file 41598_2021_3612_MOESM1_ESM.pdf]

SUPPLEMENTARY INFORMATION

For

Cuffless Blood Pressure Monitoring from a Wristband with Calibration-Free Algorithms for Sensing Location based on Bio-Impedance Sensor Array and Autoencoder

Bassem Ibrahim[1*], Roozbeh Jafari[1 2 3]

[1] Department of Electrical and Computer Engineering, Texas A&M University, College Station, TX, USA

[2] Department of Biomedical Engineering, Texas A&M University, College Station, TX, USA

[3] Department of Computer Science and Engineering, Texas A&M University, College Station, TX, USA

[*]Corresponding author: bassem.zaki@gmail.com

`

SUPPLEMENTARY NOTE 1: PULSE MORPHOLOGY VARIATION WITH BP

The proposed method of this study relies on extracting the significant features in the blood pulsatile activity in the Bio-Z signals measured from the wrist arteries that are a function of BP and can be used as the input features for the BP regression models that are trained based on the subject pulsatile properties. In order to show the change of Bio-Z pulse morphology with BP, we categorized the Bio-Z pulses into 3 groups based on BP by dividing the range of mean arterial pressure (MAP) dataset into 3 equal BP ranges of high, moderate and low MAP. The Bio-Z pulse signal (ΔBio-Z) is inverted to be proportional to blood volume changes and divided into separate heartbeat segments at the diastolic (DIA) point. The timestamps of the pulse segment for each heartbeat are normalized with respect to the heartbeat time period. The amplitude of the pulse segment is normalized by considering the DIA point as zero amplitude and dividing the amplitude of the whole heartbeat amplitude by its peak point which is the systolic (SYS) point, so that the amplitude of all pulses varies in the range of 0 to 1. In order to get the mean of the pulses for each BP group, the pulse samples are aligned in time by resampling all the heartbeat segments at the same time samples. The resampling is done by linear interpolation to get the new time samples.

The mean pulse of each BP group is plotted in Figure S1 (a). Also, the 20% and 80% percentiles of the pulses are plotted to show the spread of the pulses within each BP group. The mean pulse of the three BP groups to show the difference between pulse morphology over BP indicated by the change in the rising slope and the amplitude and time of the dicrotic notch that are used as our features for BP models to detect BP from Bio-Z signal. In addition, Figure S1 (b) plots the mean pulse for three participants to show the same trend happens for different participants but with different pulse morphology for each participant. It demonstrates the importance of using personalized BP models to capture the differences in pulse morphology that changes with BP from

one participant to another. Also, there is a significant change in pulses from beat to beat which can be solved by averaging the Bio-Z pulses through a moving average window over 20 heartbeats.

SUPPLEMENTARY NOTE 2: SENSING MODEL

In order to understand the transfer function (h), we created a model the explains the measured signal (V) as a function of the arterial pulsation. The blood pulsation in the artery causes changes in blood volume which leads to a change in impedance. The measured voltage signal is the multiplication of injected current and the tissue impedance. The amplitude of the measured pulse signal due to blood pulsation over a local segment on the artery is affected by the distance between the sensor and the artery. The pulse amplitude decreases as the distance between the sensor and the artery increases. In addition, the length of the segment that is measured on the artery is affected by the current distribution in the tissue which is maximum at the sensor location and decreases as the distance from the sensor increases. We can conclude from these observations that the relation between the measured signal $V(t)$ and the artery pulsation

The model consists of the signal $Y(t)$ that represents the blood pulsation in the artery which is the target signal for sensing. The pulse signal $Y(t)$ flows through the artery in the direction of blood flow. We can model the artery by dividing it along the blood flow direction into N elements as shown in Figure S2. Assume the signal $Y(t)$ represents the pulse signal at the first artery element (n=0) and the signal is delayed by each element with time delay t_d. Therefore, the signal at each element can be modeled as a delayed version of the original pulse $Y(t)$ and represented by $Y(t-nt_d)$ at element n. The measured signal $V(t)$ is modeled as the summation of the signals for all elements multiplied by a weight b_n that represents the effect of the tissue between the element and the sensor

and this weight decreases as the distance between the sensor and the element increases. This model can be represented mathematically by this equation

$$X(t) = b_0Y(t) + b_1Y(t - t_d) + b_2Y(t - 2t_d) + \cdots + b_{N-1}Y(t - (N - 1)t_d)$$

This is the equation of a filter with impulse response consists of the weights b_n. This shows that the transfer function (h) between the artery pulse signal (Y) and the sensor output on the skin (X) can be considered as a linear filter. Based on this conclusion, we designed the reconstruction algorithm that can estimate the linear functions h for each sensor location which leads to the estimation of the source arterial pulse signal Y as explained in the following sections.

SUPPLEMENTARY NOTE 3: BP MODEL TRAINING

Our regression model for BP estimation is **adaptive boosting** (AdaBoost) which belongs to the ensemble learning category that refers to a collection of methods that learn a target function by training a number of individual weak learners and combining their predictions. This method is used to provide a more reliable estimation of a complex problem that can be decomposed into multiple sub-problems which are easier to understand and solve.

AdaBoost is characterized by training individual classifiers on different datasets obtained by resampling a common training set. The AdaBoost model weights are estimated using Python scikit-learn library through an iterative algorithm that operates as follows:

– At iteration n, boosting provides the weak learner with a distribution D_n over the training set, where (i) represents the probability of selecting the ith example. The initial distribution is uniform: $D_1(i) = 1/N$. Thus, all examples are equally likely to be selected for the first component.

– The weak learner subsamples the training set according to D_n and generates a trained model or hypothesis H_n

– The error rate of H_n is measured with respect to the distribution D_n

– A new distribution D_n+1 is produced by decreasing the probability of those examples that were correctly classified, and increasing the probability of the misclassified examples

– The process is repeated T times, and a final hypothesis is obtained by weighting the votes of individual hypotheses $\{h_1, h_2, \ldots, h_T\}$ according to their performance

SUPPLEMENTARY NOTE 4: BIO-Z CIRCUIT DESIGN STRATEGY

The hardware is built around the ARM Cortex M4 MCU, which generates the user-defined digital AC waveform that is converted to a voltage signal by a 16-bit DAC (DAC8811, Texas Instruments, USA) as shown in Figure S7. In its turn, the DAC utilizes a negative feedback loop on a low-noise operational amplifier (OPA211, Texas Instruments, USA) to generate an AC current signal with programmable amplitude and frequency. A series capacitor at the DAC output is used to block the DC current component's injection into the human body. To extract the Bio-Z signal, we measure the voltage signal induced from the modulation of body impedance to the amplitude and phase of the injected current signal. The voltage signal is sensed by a pair of voltage electrodes and then amplified with a low-noise instrumentation amplifier (IA). A high-precision ADC samples the IA output through an analog anti-aliasing low-pass filter with a cut-off frequency of 30 kHz. The ADC (ADS1278, Texas Instruments, USA) samples the voltage at 93.75 kHz sampling frequency with a 24-bit (0.3 μV) resolution to provide enough precision. The analog front end and the MCU can handle to measure simultaneously 8 independent Bio-Z streams and various analog readings. In this study, the first 6 channels are selected for high-resolution Bio-Z sensing and the 7th channel is reserved for simultaneous PPG readings used for syncing with the Finapres NOVA BP system. The sampled data is forwarded to PC via the MCU and Hi-Speed USB Bridge for signal post-processing through MATLAB. The Bio-Z post-processing algorithms start with a bandpass filter centered at the current injection frequency to remove out-of-band noise. Then, the real and imaginary components of Bio-Z are extracted by synchronous demodulation through multiplying the measured signals by the sinewave generated from the MCU and its 90-

degree phase shift. The final Bio-Z signals are filtered by a second-order digital IIR low pass filter (LPF) with a cut-off frequency of 6 Hz. The cut-off frequency was selected to remove the carrier signal after demodulation and the out-of-band noise while keeping the main frequency components of the pulse signal which are below 6 Hz.

An interface between the hardware and the stationary computer is developed in Visual Studio so that the user can directly program the frequency and amplitude parameters of the current injection signal, and the time length of bio-impedance data acquisition. Upon instructing the parameters, the interface receives the digitized bio-impedance data transmitted by the Bio-Z XL and stores them on a local hard drive for signal processing and features extraction. The frequency of the alternating current signal can be programmed in the range of 2-20 kHz.

The quality of the Bio-Z pulsatile signal improves with increasing the amplitude of the injected current because this increases the signal-to-noise ratio. Cell membrane in tissue demonstrates capacitive properties and as a result, its impedance is inversely proportional to the current injection frequency, i.e. more current can flow inside the cells at higher frequencies. Moreover, the impedance of skin-electrode contact reduces at higher frequencies; hence, the current injected to tissue through the pair of electrodes will not suffer from saturation issue. Consequently, in trade-off with the maximum sampling rate of the Bio-Z XL, 10 kHz is chosen as the optimum frequency of the current signal. According to IEC 60601-1 standard for medical electrical equipment, in order to comply with the safety regulations, and according to the skin-electrode impedance at 10 kHz, we selected the maximum allowed current amplitude of 0.3 mA [1].

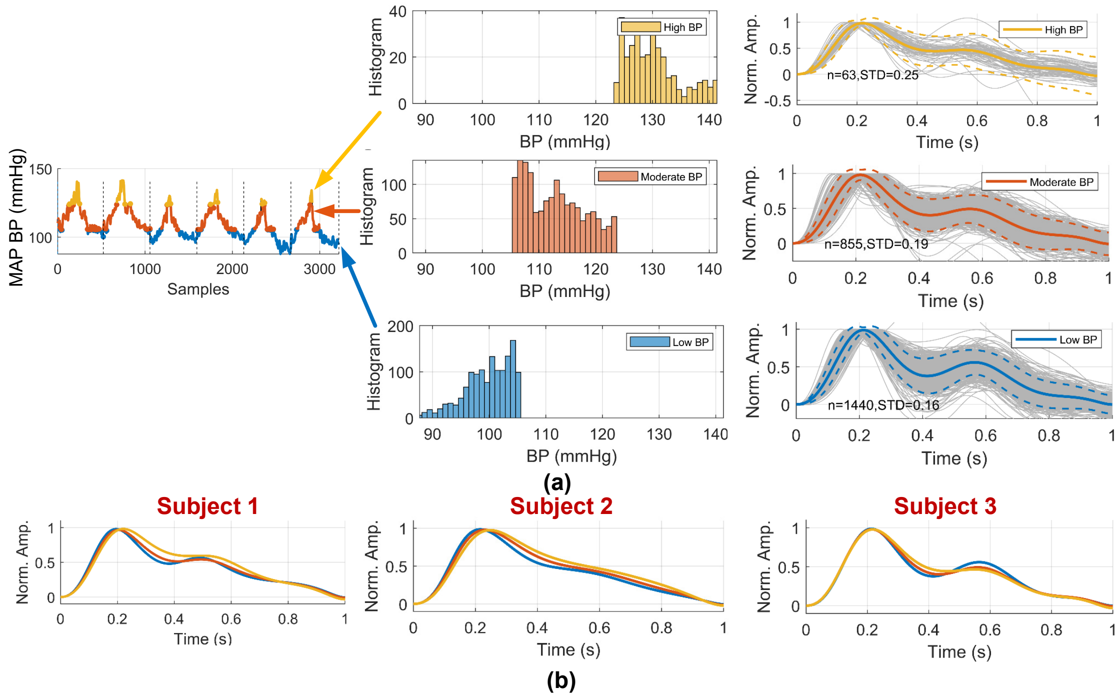

Figure S1. (a) The mean arterial pressure (MAP) of subject 1 is divided into 3 groups: low (less than 105 mmHg), moderate (from 105 to 125 mmHg) and high (above 125 mmHg) ranges showing the BP histogram for each group and the corresponding normalized Bio-Z pulses with the mean and 20 and 80 percentiles indicators. (b) The mean of bio-impedance pulses for each BP range for 3 different participants that show the variation of bio-impedance pulse morphology with BP and from one subject to another.

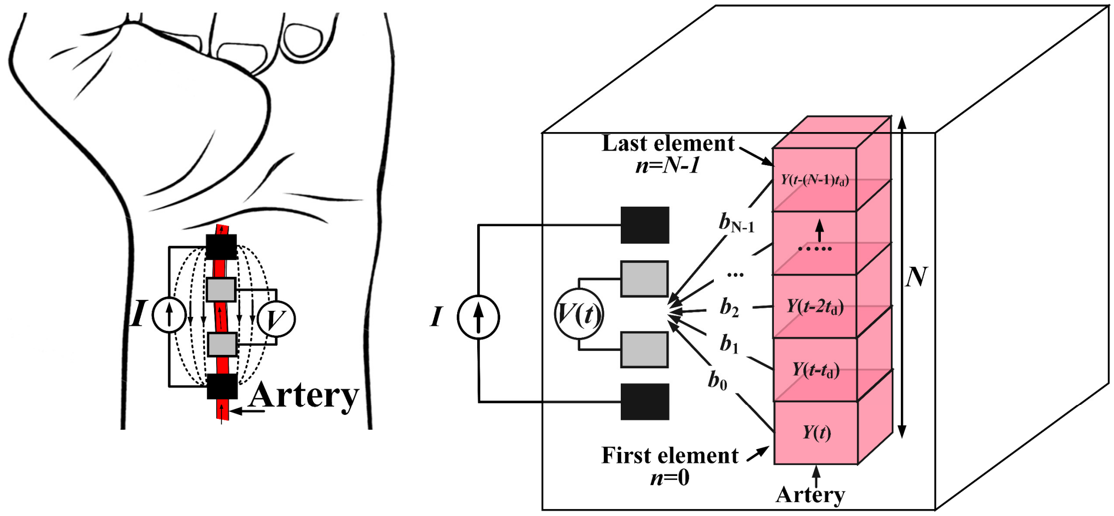

Figure S2. The Bio-Z pulsatile sensing model that represents the artery by N small segments and the pulsatile activity as $Y(t)$ that propagates in the artery with delay t_d for each artery's segment. The sensed signal $V(t)$ is the weighted sum of the pulse signal at each element which is equivalent to a linear filter for the transfer function (h).

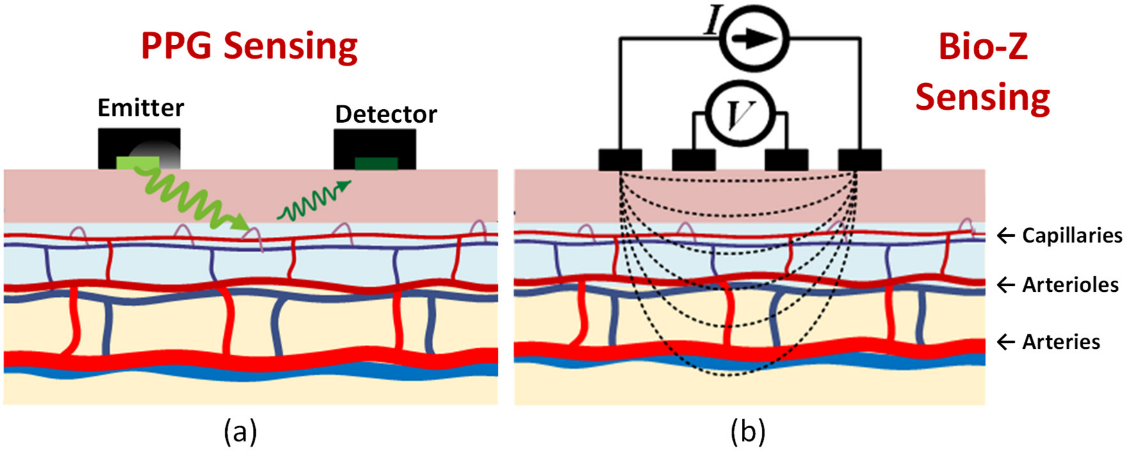

Figure S3. (a) Shallow penetration of the optical PPG sensor, which can only detect the blood pulse at the capillaries, (b) Deep penetration of the current signal of bio-impedance sensor, which reaches the arteries and detect arterial pulse wave; therefore, can provide an accurate estimation for blood pressure.

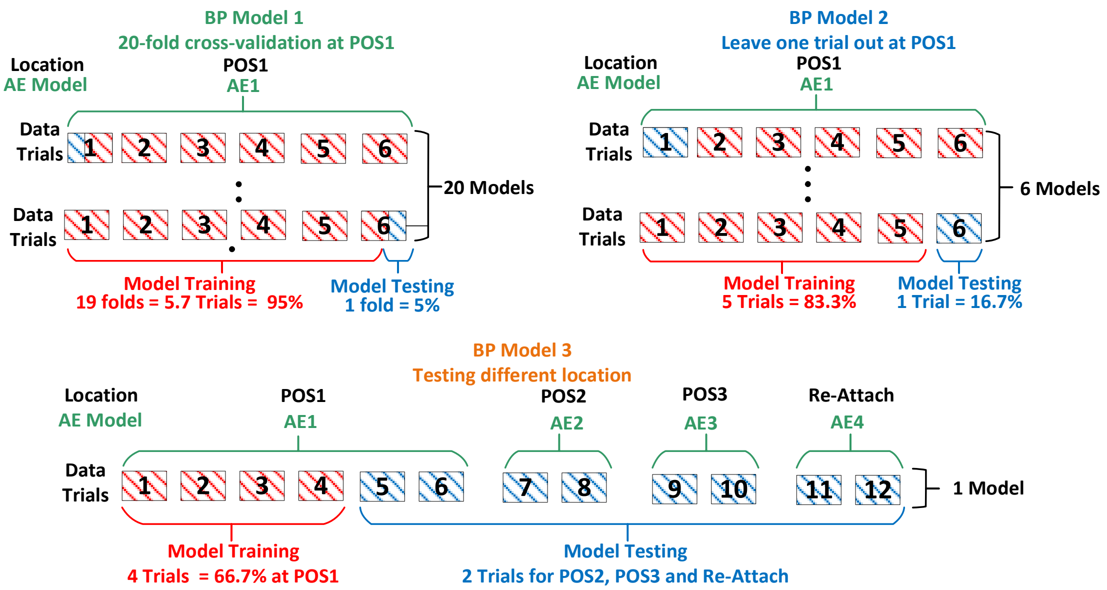

Figure S4. The visual illustration of the three training methods used in this work: 20-fold cross validation, leave one trial out and testing different location showing the split of the BP trials between training and testing data and the corresponding sensing locations and CNN autoencoder models.

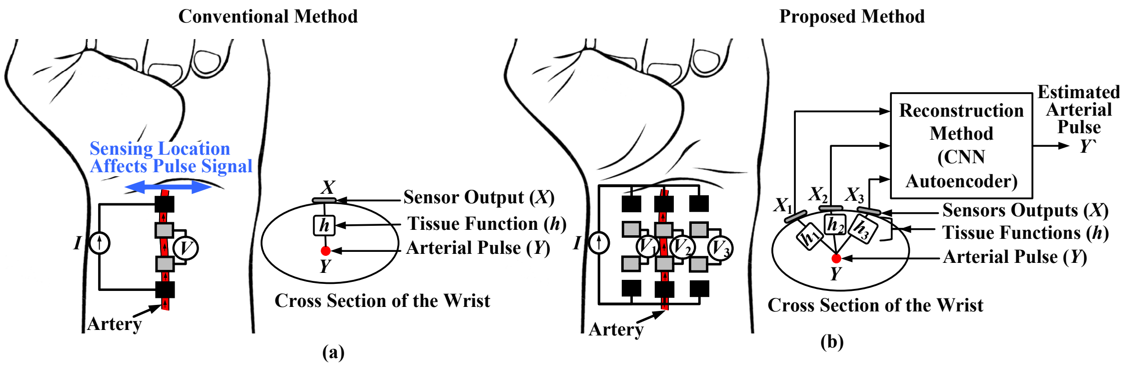

Figure S5. (a) The conventional method of measuring the pulsatile activity based on a single measurement that is affected by the sensing location and degrades BP performance. (b) the proposed method of using multi- sensor pulse signal estimation that are used for reconstruction of the arterial pulse using CNN autoencoder. This method provides accurate pulsatile activity of the artery independent on the sensing location which improves the BP estimation at different locations.

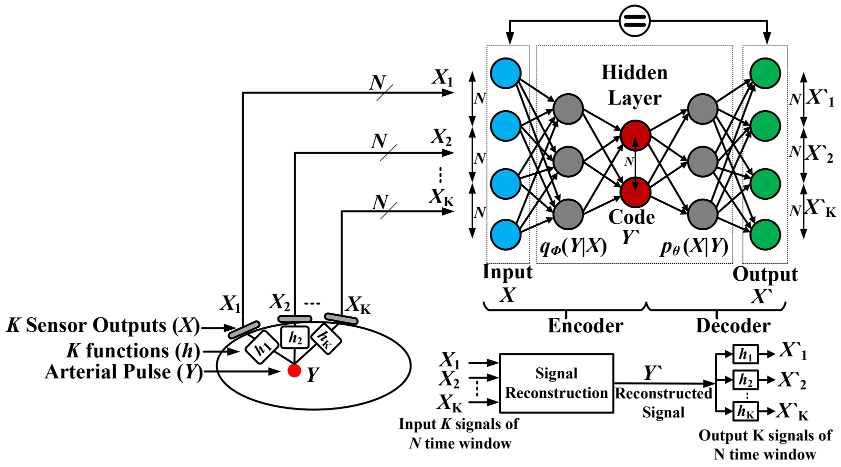

Figure S6. The proposed method of using an autoencoder to find a lower-dimension representation of the inputs from multiple sensors placed on the skin around the artery. The lower-dimension representation is the output of the encoder and represents the estimated arterial pulse signal. The decoder network is responsible of regeneration of the inputs at the output layer. The decoder network represents the linear transfer function of the artery to the sensor and the encoder represents the reconstruction function of the arterial pulse from the input observations.

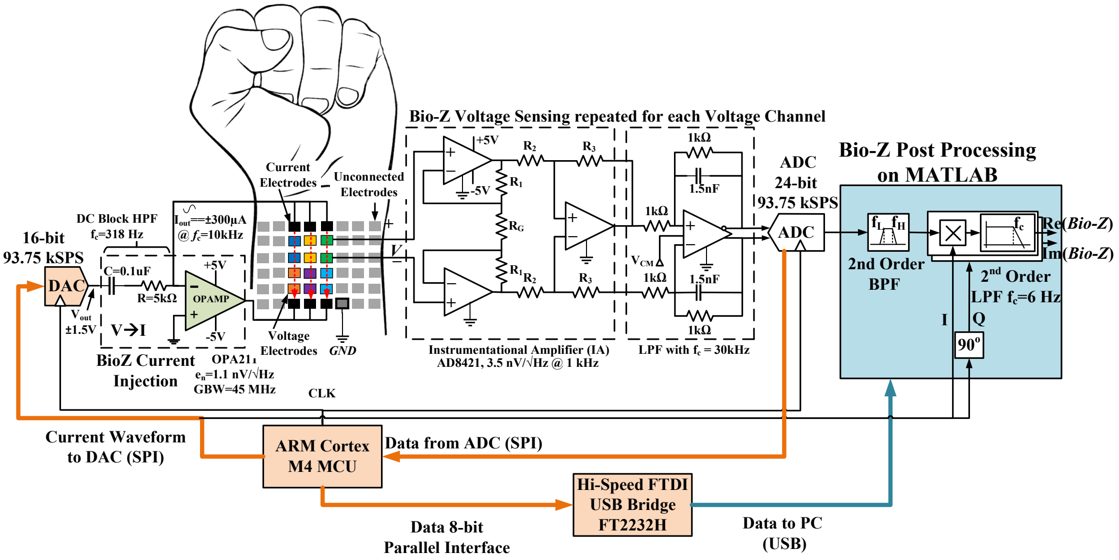

Figure S7. The block diagram of the Bio-Z sensing hardware and signal processing that is implemented in the Bio-Z XL PCB and the post-processing algorithms in MATLAB. The sensing hardware is responsible for the current injection through the top and bottom electrode row in the electrode array and voltage sensing from the 3 electrode columns through 6 Bio-Z voltage channels. The hardrwae control the sensing parameters consisting of frequency and amplitude of the current signal, and the time interval for bio-impedance data acquisition. The PC receives the digitized data and store on local hard drive. The data are then processed in MATLAB and de-mixed bio-impedance signals are extracted

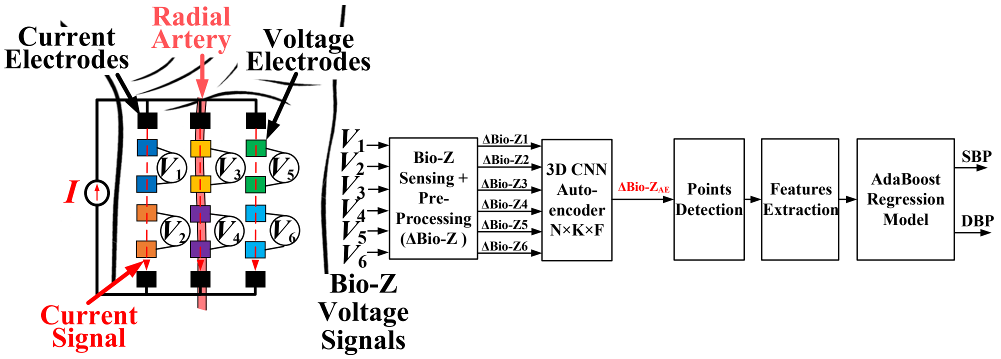

Figure S8. The wrist-worn BP monitoring system based on the Bio-Z sensor array on the wrist connected to the Bio-Z sensing hardware. The Bio-Z signal processing generate the pulse signals (ΔBio-Z) for each sensor which are used to estimate the arterial pulse signal ($\Delta Bio\text{-}Z_{AE}$) based on CNN autoencoder. The systolic BP (SBP) and diastolic BP (DBP) are predicted by AdaBoost regression model based on feature extracted from the characvterstic points of the estimated arterial pulse signal.

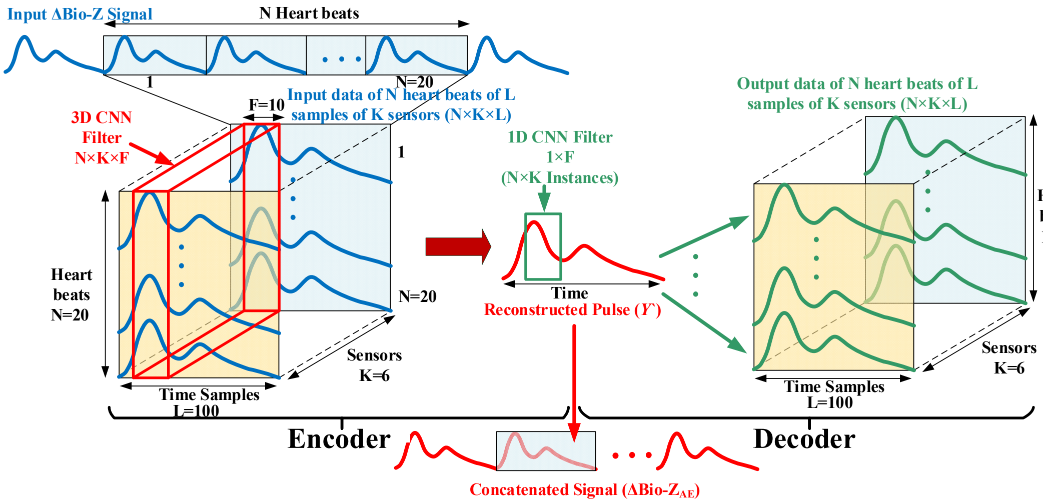

Figure S9. The description of the CNN autoencoder network structures and data arrangement. The input data are arranged in $N\times K\times L$ array and the encoder network includes a 3D filter with $N\times K\times F$ size and the decoder network 1D CNN network repeated $N\times K$ times with a convolution window size of F.

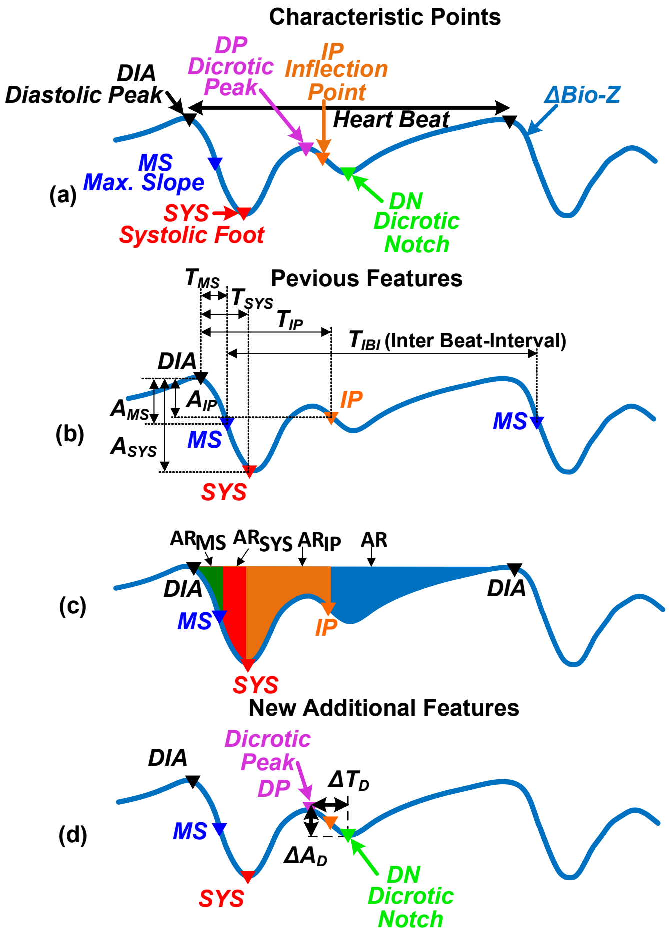

Figure S10. (a) ΔBio-Z signal over one heart beat with illustration of its six characteristic points. The points are diastolic peak (DIA), maximum slope (MS), systolic foot (SYS), inflection point (IP), dicrotic peak (DP) and dicrotic notch (DN), (b) The previous features of time and amplitude for a single bio-impedance signal from the diastolic peak to the rest of points, (c) The area features for a single bio-impedance signal from the diastolic peak to the rest of points, and (d) The new additional features proposed in this work in order to include the changes that occur in the dicrotic notch and peak points around the IP point in the BP estimation models.

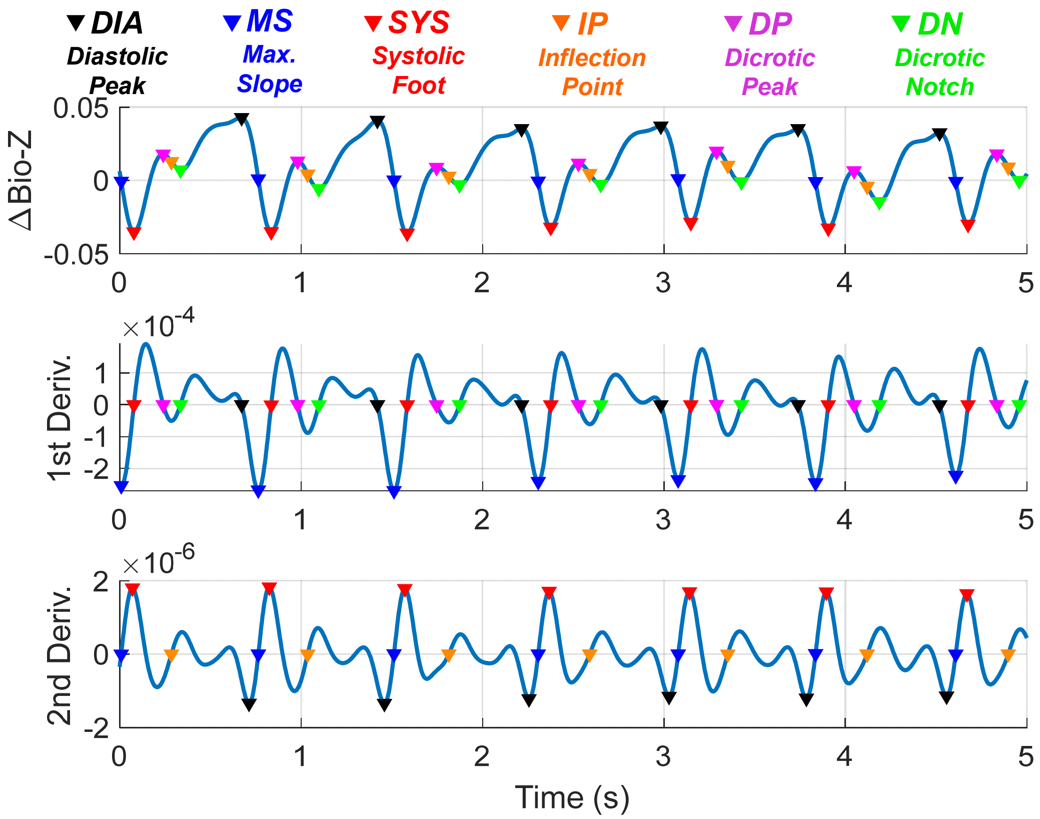

Figure S11. An example of the detection of the characteristic points of the ΔBio-Z based on the first and second derivatives from a measured Bio-Z signal. The plot shows the point detection algorithm can accurately detect all the chrematistic points for six heart beats in the presence of variations in the pulse morphology from beat to beat.

Table S1: The description of the 4 sensing locations POS1, POS2, POS3 and Re-Attach and the corresponding electrode configuration, sensing distance from the radial artery and the number of BP trails for each sensing location.

Sensing Position	Electrode Configuration (Columns Used)	Sensing Location (Distance from the center electrode column to the radial artery)	Number of BP trials
POS1 (Initial)	2,3,4	0 mm (aligned with the radial artery)	6
POS2	1,2,3	8.2 mm left to the radial artery	2
POS3	3,4,5	8.2 mm right to the radial artery	2
Re-Attach	2,3,4	Arbitrary distance within 8.2 mm left or right to the radial artery	2

Table S2: The comparison of the training methods 20-fold cross-validation, leave one trial out and testing different location in terms of the used time segments, sensing location and autoencoder model. The first method of 20-fold cross-validation trains and tests the model using POS1 data only by splitting the data into 20 folds with 19 folds (95% of POS1 data) for training and 1fold (5% of POS1 data) for testing. The 19 folds of training data are equivalent 5.7 BP trials or 42.75 min. of POS1 data. The testing data is continuous BP samples for about 70% of single BP trial or time segment of 2.25 min. of POS1 data that circulate for all the folds to cover the whole POS1 data and avoid the bias for training the model with a certain part of the data. The performance of this method is evaluated by the average of all 20 folds. The second method evaluates the BP model performance for a longer continuous time segment of POS1 data by using leave one complete trial out cross-validation method. In this method, the POS1 data is splitted into 5 BP trials of POS1 data (37.5 min.) for training data and the remaining single BP trial of POS1 (7.5 min.) for the testing data. The BP trial used for testing data loops all the six BP trials of POS1 data and the performance of this method is evaluated by the average of the 6 testing BP trials. The third method evaluates the BP performance for different sensing locations and for longer time segments by training the model with the first 4 BP trials of POS1 data (30 min.) and testing the model with the remaining 8 BP trials for the four locations (POS1, POS2, POS3 and Re-Attach) by taking the average of each 2 trial for each location which are 15 min. time segment. The arterial pulse signal (ΔBio-ZAE) is estimated for each location by four separate CNN models denoted by AE1, AE2, AE3 and AE4 for the locations POS1, POS2, POS3 and Re-Attach respectively.

BP Model Training/Testing Method	No. of Trained Models	Training Data				Testing Data			
		Time (min.)	Percentage of POS1 data	Location	AE Model	Time (min.)	Percentage of POS1 data	Location	AE Mode l
20-fold cross-validation	20	42.75	95%	POS1	AE1	2.25	5%	POS1	AE1
Leave on trial out	6	37.5	83.3%	POS1	AE1	7.5	16.7%	POS1	AE1
Testing different location	1	30	66.7%	POS1	AE1	15	33.3%	POS1	AE1
						15	33.3%	POS2	AE2
						15	33.3%	POS3	AE3
						15	33.3%	Re-Attach	AE4

Table S3: The BP estimation performance using the proposed method with the 20-fold cross validation for each subject.

Subject	Number of Samples	DBP						SBP					
		RMSE (mmHg)	R	Mean Error (mmHg)	STD Error (mmHg)	BP Mean (mmHg)	BP Range (mmHg)	RMSE (mmHg)	R	Mean Error (mmHg)	STD Error (mmHg)	BP Mean (mmHg)	BP Range (mmHg)
1	3206	5.4	0.81	0.1	5.4	73.1	42.1	5.9	0.79	0.2	5.9	137.3	46.5
2	3483	4.4	0.80	0.0	4.4	77.9	37.0	5.8	0.76	-0.3	5.8	120.1	46.9
3	3121	4.5	0.91	0.3	4.5	83.0	42.8	7.1	0.86	0.2	7.1	138.3	63.7
4	2670	5.5	0.68	0.5	5.4	82.3	41.5	7.3	0.75	0.6	7.3	137.7	55.0
Average	3120	5.0	0.80	0.2	5.0	79.1	40.8	6.6	0.79	0.2	6.5	133.3	53.0
STD	292	0.5	0.08	0.2	0.5	4.0	2.3	0.7	0.04	0.3	0.7	7.6	7.0

Table S4: The average BP estimation performance for all subjects using the 20-fold cross validation and the proposed method compared to the baseline method.

The Method	DBP				SBP			
	RMSE (mmHg)	R	Mean Error (mmHg)	STD Error (mmHg)	RMSE (mmHg)	R	Mean Error (mmHg)	STD Error (mmHg)
Baseline	6.6	0.64	-0.1	6.6	9.0	0.57	-0.2	9.0
AE	6.0	0.71	0.1	6.0	8.1	0.67	0.2	8.1
AE + New features	5.7	0.74	0.2	5.7	7.5	0.73	0.2	7.5
The Proposed Method (AE + New features + BP avg.)	5.0	0.80	0.2	5.0	6.6	0.79	0.2	6.5

Table S5: The subject average BP estimation performance for the leave one trial out cross validation using the proposed method compared to the baseline method.

The Method	DBP				SBP			
	RMSE (mmHg)	R	Mean Error (mmHg)	STD Error (mmHg)	RMSE (mmHg)	R	Mean Error (mmHg)	STD Error (mmHg)
Baseline	6.8	0.63	-0.1	6.8	9.1	0.57	-0.2	9.0
AE	6.3	0.67	0.2	6.3	8.2	0.66	0.1	8.2
AE + New features	5.9	0.72	0.3	5.8	7.8	0.71	-0.1	7.8
The Proposed Method (AE + New features + BP avg.)	5.2	0.77	0.3	5.2	6.9	0.76	-0.1	6.8

Table S6: The DBP and SBP error distribution for the three BP error ranges under the thresholds 5 mmHg, 10 mmHg and 15 mmHg according to BHS standard for the leave one trial out cross validation using the proposed method.

		≤5 mmHg	≤10 mmHg	≤15 mmHg
**Our Result**	**DBP**	**69%**	**94%**	**99%**
	**SBP**	**60%**	**86%**	**96%**
**BHS**	**Grade A**	**60%**	**85%**	**95%**
	**Grade B**	**50%**	**75%**	**90%**
	**Grade C**	**40%**	**65%**	**85%**

Table S7: The subject average BP estimation performance using the proposed method compared to the baseline method for different locations

.

Testing Location		Baseline							
		DBP				SBP			
		RMSE (mmHg)	R	Mean Error (mmHg)	STD Error (mmHg)	RMSE (mmHg)	R	Mean Error (mmHg)	STD Error (mmHg)
Same Location	POS1	7.2	0.67	-2.9	6.5	8.2	0.64	1.0	8.1
Diferent Location	POS2	8.9	0.56	-2.2	6.9	10.2	0.52	0.5	9.1
	POS3	8.8	0.46	-0.1	8.0	12.3	0.43	3.6	10.6
	Re-Attach	10.6	0.49	-4.1	8.6	11.7	0.47	0.7	9.9
	Average	9.4	0.50	-2.1	7.8	11.4	0.47	1.6	9.9

Testing Location		Proposed Method							
		DBP				SBP			
		RMSE (mmHg)	R	Mean Error (mmHg)	STD Error (mmHg)	RMSE (mmHg)	R	Mean Error (mmHg)	STD Error (mmHg)
Same Location	POS1	5.8	0.79	-1.9	5.3	6.2	0.85	2.2	5.6
Diferent Location	POS2	6.8	0.74	-2.1	5.1	8.9	0.67	1.2	7.4
	POS3	7.1	0.75	2.2	6.0	11.5	0.67	6.7	8.5
	Re-Attach	8.2	0.68	-4.1	6.9	10.3	0.52	3.1	9.6
	Average	7.4	0.72	-1.3	6.0	10.2	0.62	3.7	8.5

Table S8: The CNN autoencoder parameters

CNN autoencoder parameters	Description	Value
N	Number of heart beats	20
K	Number of sensors	6
F	Filter width	10
L	Number of samples in heart beat	100
$2 \times N \times K \times F$	Number of weights	2,400

Table S9: Wrist Bio-Z features

Feature Set	Feature Description	Number of Features
Time	The time interval from the DIA point to the MS, SYS and IP points, which are T_{MS}, T_{SYS} and T_{IP}, as shown in Figure S10 (b), normalized by T_{IBI}.	3
Amplitude	These are the difference in amplitude from DIA point to the MS and IP points, which are A_{MS} and A_{IP} as shown in Figure S10 (b), normalized by the pulse foot to peak amplitude A_{SYS}	2
Area	The areas under the ΔBio-Z curve starting from the DIA point to the MS, SYS and IP of points, which are AR_{MS}, AR_{SYS}, AR_{IP} normalized by full pulse area AR as shown in Figure S10 (c). The area under the curve represents the total peripheral resistance.	3
Dicrotic	New added features for the dicrotic peak to notch amplitude ΔA_D and time ΔT_D normalized by A_{SYS} and T_{IBI} respectively as shown in Figure S10 (d).	2
Histogram	The population of 5 amplitude bins which are the division of the normalized pulse amplitude into 5 equal intervals.	5
Total		15

References

[1] "Medical electrical equipment, Part 1: General requirements for basic safety and essential performance, ANSI/AAMI ES60601-1:2005/A1:2012." ANSI/AAMI ES60601-1:2005/A1:2012. (accessed.
